# Effectiveness of improving nutrition on depressive symptoms and work ability: Study protocol for the mind nutrition randomized controlled trial

**DOI:** 10.1177/02601060251332358

**Published:** 2025-04-21

**Authors:** Aino Kipfer, Minna Kahala, Henna Kyhä, Cimmo Nurmi, Juha Puustinen, Jyrki Korkeila, Anu Ruusunen, Susanna Kunvik

**Affiliations:** 1 52915 Research Center for Human Functioning, Satakunta University of Applied Sciences, Pori, Finland; 2 Institute of Public Health and Clinical Nutrition, University of Eastern Finland, Kuopio, Finland; 3 Satasairaala, Unit of Neurology, 637679 Satakunta Welfare Area, Pori, Finland; 4 60654 Faculty of Medicine, Department of Psychiatry, University of Turku, Turku, Finland; 5 Wellbeing Services County of North Savo, Mental Health and Wellbeing, Kuopio University Hospital, Kuopio, Finland; 6 Institute for Mental and Physical Health and Clinical Translation (IMPACT), Food & Mood Centre, School of Medicine, Deakin University, Geelong, Australia

**Keywords:** Nutrition, depressive symptoms, nurses, work ability, eating behaviour, quality of life

## Abstract

**Background:** Depression is a growing public health concern that negatively impacts work ability and performance. Psychiatric nurses have an elevated risk for depressive symptoms, and it is essential to ensure their good mental health and work ability. The association between nutrition and depression is recognized, but there is a need for randomized, controlled intervention studies investigating the effectiveness of diet on depressive symptoms and related work impairment. **Aim:** Mind Nutrition is a randomized, controlled intervention study examining whether improving nutrition can reduce depressive symptoms and depressive symptoms-related sick leaves and enhance work ability and quality of life among mental health and substance abuse professionals. **Methods:** We will recruit 125 nurses and social welfare professionals from the mental health and substance abuse field of the Satakunta Wellbeing Services County. Half of the participants will be randomized to the intervention and half to the control group. Subjects in the intervention group will participate in two individual and three group counselling sessions during a 6-month intervention. Nutrition counselling follows the Finnish Nutrition Recommendations. The control group will maintain their habitual diet. The Center for Epidemiological Studies – Depression, depressive symptoms-related sick leave days, two work ability indicators, a Food Frequency Questionnaire, three-day food records, Three-Factor Eating Questionnaire – 18 and WHO-8 Eurohis Quality of Life will be assessed at baseline and 6 months. **Summary:** This study will provide evidence of the effectiveness of nutrition counselling on depressive symptoms and related sick leave days, work ability and quality of life.

## Introduction

Depression is a common mental disorder, with around 280 million people in the world suffering from it (World Health Organization [WHO], 2023). It negatively affects the quality of life, including social life, emotional and physical well-being and work life. At the societal level, depression causes a substantial economic burden. It is associated with greater work absenteeism, short-term disability, decreased productivity and presenteeism (Brandford and Reed, 2016). The Organisation for Economic Co-operation and Development (OECD, 2018) has estimated the total costs of mental health problems in Europe to be more than EUR 600 billion yearly which is more than 4% of the gross domestic product.

Treating depressive symptoms at an early stage is crucial for preventing their aggravation (Kessler et al., 2003). Nurses have a two-fold risk of depression compared to those in other professions (Brandford and Reed, 2016), with mental health nurses experiencing particularly high levels of depressive symptoms (Brandford and Reed, 2016; Opoku Agyemang et al., 2022; Tsaras et al., 2018). Recommendations for prevention include lifestyle interventions, focusing on exercise, healthy diet, social relationships and adequate and good quality sleep (Finnish Current Care Guidelines for Depression, 2024; World Health Organization, 2023). There is convincing evidence for the link between the Mediterranean diet (Serra-Majem et al., 2020) and a lower dietary inflammatory index and a lower risk of depression (Gianfredi et al., 2023). The relationship between diet and depression is likely bidirectional. The biological mechanisms linking nutrition to depression include, for example, gut microbiota, low-grade inflammation, epigenetic changes and oxidative stress (Marx et al., 2021).

Mediterranean diet interventions, utilizing either individual or group counselling, have significantly improved moderate to severe depressive symptoms (Bayes et al., 2022; Francis et al., 2019; Jacka et al., 2017; Parletta et al., 2019), with two studies demonstrating good results in cost-effectiveness analyses (Chatterton et al., 2018; Segal et al. 2020). The studies have reported a reduction in depression to mild or sub-clinical levels (Bayes et al., 2022; Francis et al., 2019; Jacka et al., 2017; Parletta et al., 2019) and an association between improved diet and reduced depression (Francis et al., 2019; Jacka et al. 2017; Parletta et al., 2019). Other intervention studies are predominantly based on secondary analyses and are heterogeneous regarding depression definition and methods (Firth et al., 2019; O’Neill et al., 2022). The MooDFOOD depression prevention study (Bot et al., 2019) using nutrition-focused behavioural therapy, multi-nutrient supplement or a combination found positive effects only among active participants and those with high baseline depressive symptoms.

To our knowledge, the effects of nutrition on depression-related work disability have not been researched before. One study found associations between dietary factors and burnout symptoms, indicating that nutrition does have a role in coping at work (Penttinen et al., 2021). Overall, lifestyle interventions have shown their potential to improve the work ability and productivity of the workforces (Grimani et al., 2019). People with a high depression risk should be a key target group for interventions to reduce work disability (Wedegaertner et al., 2013). Nutrition interventions could reduce the economic burden of depression by also preventing physical comorbidities of depression, such as obesity (Luppino et al., 2010), type 2 diabetes (Semenkovich et al., 2015) and cardiovascular diseases (Krittanawong et al., 2023).

The primary aim of the Mind Nutrition randomized controlled trial is to examine the effectiveness of nutrition counselling on depressive symptoms and work ability among nurses and social services professionals working in the mental health and substance abuse fields. The secondary aims are to examine the effectiveness of depression-related sick leave days, quality of life and dietary habits. This article is a detailed description of the study protocol. This is the first study that provides evidence on the effects of nutritional counselling on depressive symptoms, work absenteeism and work ability among mental health professionals.

## Methods

### Participants and recruiting

#### Participants

We will recruit 125 licensed practical nurses, registered nurses, mental health nurses and social services professionals in the mental health and substance abuse fields in Pori, Satakunta Wellbeing Services County, Western Finland. We will recruit participants working in special care and primary care, outpatient departments, institutional care and rehabilitation services. The inclusion and exclusion criteria are as follows:

*Inclusion criteria:*

1. Aged 18 years or over
2. Adequate Finnish language skills
3. Work-capable
4. Currently employed by Satakunta Wellbeing Services County for at least 6 months
5. Works in the mental health and substance abuse fields
6. Education is in one of the following areas: licensed practical nurse, registered nurse, mental health nurse or has a bachelor's degree in social services
7. Agrees to commit to either of the groups (intervention/control) during the 6 months of intervention
8. Able to give informed consent.

*Exclusion criteria:*

1. Acute mental health symptoms and inability to work
2. Terminal phase disease or unstable severe chronic disease (consultation with the responsible physician)
3. Employed for a shorter period than 6 months
4. History of eating disorder (consultation with the responsible physician)
5. A score of ≥16 on the CES-D (Center for Epidemiological Studies – Depression) scale (Radloff, 1977) and reduced work capacity (determined in consultation with the medical expert in mental health)
6. Substance abuse based on the Alcohol Use Disorder Identification Test – Consumption (AUDIT-C) score (consultation with the responsible physician)

#### Sample size calculation

We calculated the sample size based on the expected change in depressive symptoms, measured with the CES-D scale (Radloff, 1977). We expect depressive symptoms to decrease in the intervention group from 14 to 9.9 points (Katsuta et al., 2021; Konttinen et al., 2010; Mondal et al., 2022) and assume that depressive symptoms will not change in the control group. The calculation is based on an estimated power of 0.8 with an expected dropout rate of 25%. The calculated sample size was 125, meaning 62 or 63 participants per group (ß = 0.2, a 0.05).

#### Recruitment

**Figure 1.**
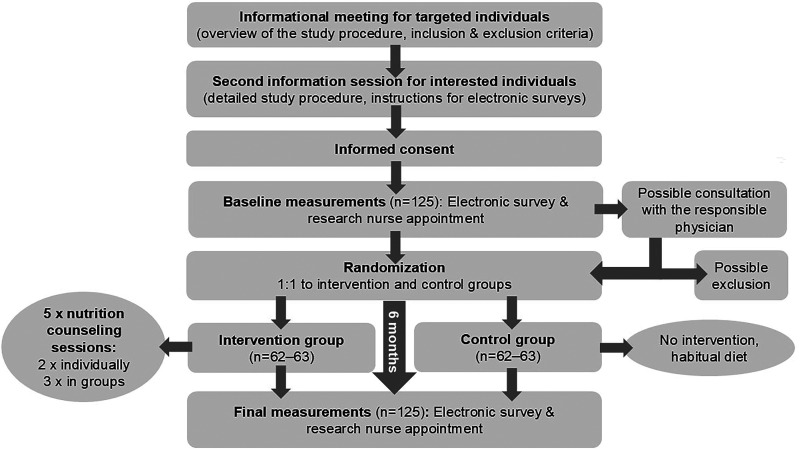
Flow chart of the study.

We will organize an informational online meeting for targeted participants in which we will explain the study procedure (Figure 1). Interested participants can register for the second informational meeting, consisting of a detailed description of the study, the inclusion and exclusion criteria and the instructions for filling in the questionnaires and food records. The principal researcher will evaluate the eligibility of the interested participants who can give informed consent at the end of the second meeting. Subsequently, we will schedule individual appointments with a research nurse and ask the participants to fill in the questionnaires before the appointments. The research nurse will measure blood pressure and weight, check the questionnaires and confirm whether the subject is eligible to participate. If the questionnaires reveal something that falls under the exclusion criteria, the research nurse can consult with the responsible physician. Exclusion of the participant is possible at this point.

### Randomization

After baseline measurements and confirming that they meet the inclusion criteria, the participants will be randomly allocated in a 1:1 ratio into the intervention or control groups according to a computer-generated, blocked randomization list. A person unrelated to the study will conduct the randomization. For the participants in the intervention groups, the 6-month nutrition intervention period starts on the first day of nutritional counselling, whereas for the participants in the control group, it starts on the day randomization will be conducted. The control group will not receive any intervention but will continue their habitual diet. We will offer the control group one nutrition counselling session after the end of the intervention.

### Measurements

#### Background measurements

Background information will be collected before the randomization, utilizing the electronic survey data, and the weight and blood pressure measured at the research nurse appointments. The electronic survey will have questions about family relations, education, basic employment information and lifestyle habits, including the AUDIT-C (Saunders et al., 1993). The research nurse appointments will be organized at the university. The nurse will check the electronic questionnaire, blood pressure and weight during these appointments. We will calculate body mass index based on the measured weight and participants’ self-reported height. Although the primary focus of our intervention is not on body weight loss, we will measure it to consider the possible effect that weight loss could have on depression scores (Patsalos et al., 2021).

#### Primary and secondary outcomes

Table 1 presents the primary and secondary outcomes. The outcomes will be assessed utilizing the electronic survey at baseline and after 6 months.

**Table 1.** Primary and secondary outcomes.

Primary outcome variables	Measurement
Depressive symptoms	CES-D (Radloff, 1977)
Work ability	Current work ability compared with the lifetime best (Tuomi et al., 2007) Three-level work ability indicator (Gould et al., 2015)
Secondary outcome variables	Measurement
Sick leave days related to depressive symptoms	Structured question about sick leave days during the past 6 months
Quality of life	WHO8-EUROHIS (WHO, Power, 2003)
Dietary habits	Food Frequency Questionnaire (Männistö et al., 1996) Healthy Diet Index (HDI; Lindström et al., 2021) Three-day food records Three Factor Eating Questionnaire – 18 (TFEQ-18; Karlsson et al., 2000)
Intervention adherence	Goal Attainment Scaling (GAS; Kiresuk and Sherman, 1968) Level of participation
Intervention acceptability	The Theoretical Framework for Acceptability (TFA; Sekhon et al., 2022)

CES-D: Center for Epidemiological Studies – Depression; TFEQ-18: Three Factor Eating Questionnaire – 18; WHO: World Health Organization.

##### Primary outcome: Change in depressive symptoms

The primary outcome is the change in depressive symptoms measured with the CES-D scale (Radloff, 1977). This scale is commonly used to assess depressive symptoms in the general population. Its 20 items enquire about various symptoms of depression experienced over the past week, such as feelings of sadness, loss of interest and sleep disturbances. Each item is scored on a scale from 0 to 3, indicating the frequency of experiencing that symptom (0 = Rarely or none of the time, 1 = Some or a little of the time, 2 = Occasionally or a moderate amount of time, 3 = Most or all the time). Points from questions 4, 8, 12 and 16 are reverse-scored. The total CES-D score is the sum of the responses to all items, between 0 and 60, with a higher score reflecting more severe depressive symptoms. Typically, a cut-off score of 16 or more points indicates significant depressive symptoms.

##### Primary outcome: Change in work ability

We will measure work ability with two indicators. The first one is ‘Current work ability compared with the lifetime best’ assessed with one question: ‘Assuming that the best working capacity you have ever had would score 10 on a scale of 0 to 10, how would you score your working capacity at present? A score of 0 would mean that you are completely unable to work at present’ (Tuomi et al., 2007).

The second is a three-level work ability indicator based on one question: ‘Regardless of whether you are employed or not, please estimate your current work ability. Are you: 1) completely fit for work, 2) partially unable to work and 3) completely unable to work’ (Gould et al., 2015). The first indicator has been demonstrated to be a reliable and practical alternative to the longer version of the Work Ability Index, and the second indicator has predicted the risk of retiring on disability pension (Gould et al., 2015).

##### Secondary outcome: Change in sick leave days related to depressive symptoms

We will measure depression-related sick leave days during the past 6 months. The question has the following response options: No days, 0–6 days, 7–14 days, 15–29 days, 30–49 days and 50 or more days.

##### Secondary outcome: Change in quality of life

We will assess the quality of life with the WHO8-EUROHIS questionnaire, originally developed as a part of the European Health Interview Survey (Power, 2003). It consists of four dimensions of quality of life: mental, physical, social and environmental, and it has good internal consistency and acceptability across different countries (Schmidt et al., 2006). Its eight items assess perceived health, energy levels, performance of daily activities, satisfaction with relationships, financial status and living conditions. Respondents answer the questions on a 5-point Likert scale, ranging from ‘not at all’ to ‘completely’, for instance. The mean score is obtained by adding all the item scores and dividing the sum by eight.

##### Secondary outcome: Change in dietary habits

We will evaluate dietary patterns using three-day food records and a 163-item Food Frequency Questionnaire (FFQ). The three-day food recording will utilize the Eat@Work mobile application (Jyräkoski et al., 2023), tailored to keep a reliable food diary with visual and written descriptions of the foods and beverages consumed. We will ask the participants to record their food and beverage intake for one day during the weekend and two weekdays (Thursday to Saturday or Sunday to Tuesday). The food records include the quality and quantity of the consumed foods and beverages, meal timing and additional contextual information. The research nutritionist will review the food records and request clarifying information when necessary. We will calculate the energy and nutrient intakes utilizing the Fineli National Food Composition Database in Finland (Reinivuo, 2014).

The 163-item FFQ is a slightly modified version of the FFQ used in the Kuopio Breast Cancer Study (Männistö et al., 1996), where its validity and reliability have been described. The FFQ was designed to cover the whole diet over the last 3 months. Food items are presented under 13 sub-groups, such as dairy products, grain products, vegetables, fruits and berries. The portion sizes are fixed and specified using natural units (e.g., slice, glass or grams). The FFQ comprises nine frequency options ranging from ‘never’ to ‘six or more times a day’. The average daily food consumption will be calculated by multiplying the consumption frequency by portion size (grams). Average daily energy and nutrient intakes will be calculated using the national food composition (Fineli) database. We will analyse the intake of energy, protein, fats, carbohydrates, fibre, vitamins and minerals.

The Healthy Diet Index (Lindström et al., 2021) will be calculated based on the FFQ data. It includes seven domains: meal pattern, fruit and vegetables, grains, fish and meat, dairy, fats, snacks and treats. Possible scores range from 0 to 100 points, with higher scores indicating better diet quality. It has been shown to correlate with anthropometric measurements, nutrient intakes and metabolic risk factors.

##### Secondary outcome: Change in eating behaviour

We will assess eating behaviour with the Three Factor Eating Questionnaire – 18 (Karlsson et al., 2000). Its 18 questions measure three domains of eating behaviour: emotional eating, uncontrolled eating and cognitive restraint. There is substantial evidence of a positive correlation between higher emotional eating and depressive symptoms (Dakanalis et al., 2023), and the two other subscales also have associations with depressive symptoms (Paans et al., 2018; Rachubińska et al., 2024). Responses to 17 questions are on a 4-point Likert scale, and one response is on an 8-point continuous scale. The total scores of each dimension are calculated by a formula that gives the total scores between 0 and 100 (The Finnish Association for the Study of Obesity, 2024).

##### Secondary outcomes: Intervention adherence and acceptability

We will measure intervention adherence with Goal Attainment Scaling (GAS; Kiresuk and Sherman, 1968) and the number of sessions attended during the intervention. Level of participation is the most common way of measuring adherence in psychological therapy, behavioural change and rehabilitation interventions (Giovanazzi et al., 2022). With the GAS method, participants will set goals and evaluate their achievements during individual sessions. The GAS method has been used in various healthcare interventions, and its purpose is an orientation towards participant-centred care, meaning that the focus is on what matters most to the participant (Logan et al., 2022). The goals are weighted and assigned on a 5-point scale, 0 meaning the expected, realistic outcome, +1 and +2 better than expected and −1 and −2 worse than expected outcomes. We will use a formula to calculate a T score on the attainment level of the goals (Logan et al., 2022).

##### Acceptability

We will measure the level of intervention acceptability with Sekhon's theoretical framework of acceptability (Sekhon et al., 2022) that delineates acceptability across seven dimensions – affective attitude, burden, ethicality, intervention coherence, opportunity costs, perceived effectiveness and self-efficacy – each accompanied by pertinent questions. We will use the adapted and translated version of the generic questionnaire to measure acceptability and obtain feedback. Table 2 summarizes all the measurements and time points.

**Table 2.** All measurements and time points.

	Baseline measurements		Final measurements at 6 months		Others
	Electronic survey	Research nurse appointment	Electronic survey	Research nurse appointment	
Background information	X	X			
Health information	X	X	X	X	
Blood pressure		X		X	
Weight		X		X	
Depressive symptoms (CES-D)	X		X		
Work ability (two indicators)	X		X		
Sick leave days	X		X		
Quality of life (WHO8-EUROHIS)	X		X		
Diet quality (Food Frequency Questionnaire)		X		X	
Nutrient intake (3-day food records): Healthy Diet Index (HDI)	X^1^		X^1^		
Eating behaviour (TFEQ-18)	X		X		
Goal attainment (GAS): During the intervention					X
Intervention adherence (during the intervention)					X
Acceptability (TFA)			X		

CES-D: Center for Epidemiological Studies – Depression; GAS: Goal Attainment Scaling; TFA: Theoretical Framework of Acceptability; TFEQ-18: Three Factor Eating Questionnaire – 18; WHO8-EUROHIS: World Health Organization EUROHIS.

^1^ Eat@Work mobile app.

### Intervention

Subjects in the intervention group participate in a 6-month multifactorial, tailored nutritional counselling consisting of two individual sessions with a personal nutrition plan described below and three group counselling sessions in smaller groups with 15–20 individuals. Combining individual and group counselling allows participants to meet their personal needs, set individual goals and receive peer support. The sessions are held monthly at participants’ workplaces or at the university. Table 3 presents the themes and content of the sessions in chronological order. The principles of nutrition counselling are based on the Finnish Nutrition recommendations (Finnish National Nutrition Council and Finnish Institute for Health and Welfare, 2024). We will advise the participants on the following while taking into consideration their individual needs:

1. Vegetables, fruit and berries: at least 500–800 g daily in total
2. Whole grain products: at least 90 g daily, meaning, for example, three slices of bread, one portion of oatmeal and one decilitre of cooked barley groats
3. Unsweetened, fat-free or low-fat dairy products: 350–500 mL daily
4. Non-tropical vegetable oils and vegetable-oil-based margarine: at least 25 g daily, avoiding butter and tropical oils
5. Fish: 300–450 g weekly, including at least 200 g of fatty fish
6. Pulses and legumes: 50–100 g (cooked), to replace meat and poultry, at least partly
7. Red meat: maximum of 350 g (cooked) weekly and avoidance of processed meat
8. Maximum of one egg daily
9. Water is the main drink
10. Added sugar as little as possible (maximum of 10% of energy intake)

**Table 3.** Content of the nutrition counselling sessions.

	Theoretical framework	Content	Session and home assignments
**1^st^ individual session:** Personal nutrition plan	Goal attainment scaling (GAS; Kiresuk and Sherman, 1968)	Evaluation and feedback on the participants’ diet	Personal goal setting
**1^st^ group session:** Everyday life and meal frequency	Psychoeducation	Introduction theme: Diet and depression Theme: Importance of regular meal frequency	Daily factors associated with own mental well-being Home: Test of dietary habits
**2^nd^ group session:** Good food, better mood	Peer support (Lim et al., 2021) Mindful eating (Nelson, 2017)	Notes of dietary habits: pros and cons Theme: Health-promoting diet, topic requests of the participants Mindful eating activity	Home: Adding a mind-friendly food item to one's diet Home: Daily life lists of futilities, successes and reasons for gratitude
**2^nd^ individual session:** Goal attainment and further plan	GAS (Kiresuk and Sherman, 1968) Motivational interviewing (Miller, 1996)	Discussion about goal attainment, motivation and challenges	Evaluation of goal attainment
**3^rd^ group session:** Hope for the future	Psychology of long-term lifestyle changes (Teixeira et al., 2015)	Theme: Successful eating regulation skills	Successes during the intervention

#### Individual counselling

The main contribution of the intervention is the individually tailored nutrition counselling provided to each participant by the same research nutritionist. The focus is on the personal goal towards the adoption of the recommended diet by participants through personalized goal setting. The first individual counselling session lasts 1–1.5 h. During the first session (Table 3), the nutritionist evaluates the participant's background factors, dietary habits, nutrient intake and eating behaviour. The participant sets adequate goals and means to achieve them with the nutritionist. The framework is the GAS method (Kiresuk and Sherman, 1968). Goals and means will be written in the personal nutrition plan. During the second individual meeting (1 h), the achievement of the goals will be assessed using the GAS method (Kiresuk and Sherman, 1968) and potential challenges and solutions will be discussed by applying the principles of motivational interviewing (Miller, 1996).

#### Group counselling

Participants will attend three nutrition-focused group counselling sessions, each lasting 1–1.5 h (Table 3). Each session will have its own theme and assignments for the meetings and homework. Some tasks are modified versions of those used in the StopDia diabetes prevention study (Pihlajamäki et al., 2019). Participants will receive a workbook containing the assignments and informational material. The first group session theme addresses daily routines and benefits of a regular meal frequency and includes a brief introduction to the background of the Mind Nutrition study. Participants can also request personally meaningful topics that will be covered in subsequent meetings.

The second group session focuses on the principles of a health-promoting diet. Participants share their health-promoting and unfavourable habits and receive peer support, fostering a sense of connection, sharing novel ideas and empowerment from others. The theme will be covered in a healthy diet lecture, considering the requests of the participants. The first home assignment – adding a ‘mind-friendly food item’ – means testing a new food related to a lower risk of depression, such as fish or vegetables high in folate. The purpose of the ‘daily life lists’ assignment is to scrutinize daily routines and time management as factors influencing eating habits.

The last group session directs attention towards the future. Participants will assess what they have learned and how they have succeeded during the intervention. The final theme addresses eating patterns and behavioural skills.

### Statistical analyses

The results will be analysed using the IBM SPSS Statistics program and the STATA program. A statistician unrelated to the study will perform the statistical analyses. The analyses will utilize the intention-to-treat principle (Soares and Carneiro, 2002).

The normality of variables will be examined graphically and using the Shapiro–Wilk test. For the descriptive statistics, comparisons between groups will be analysed using the independent samples *t*-test for continuous, normally distributed variables, the chi-squared test for categorical variables and the Mann–Whitney *U* test for non-normally distributed variables. The difference between groups in the change in depressive symptoms, quality of life and diet quality is continuous variables and will be analysed with repeated analysis of variance. The difference between groups in the change in work ability and sick leave days related to depressive symptoms is ordinal variables and will be analysed with mixed-effect models for ordinal data. The main outcomes will be examined first overall and then stratified by gender to assess potential gender differences in intervention effectiveness.

## Summary

Depression causes work disability and impairs the quality of life (World Health Organization, 2023). There is substantial evidence of the association between nutrition and depression, but there is a lack of research on intervention studies on depressive symptoms among high-risk groups. Based on epidemiological evidence, a healthy diet can potentially reduce depressive symptoms (Gianfredi et al., 2023). Lifestyle interventions have shown benefits for working capacity and productivity (Grimani et al., 2019). As a risk group for depressive symptoms and related work disability, mental health nurses are a crucial target group for nutrition trials (Brandford and Reed, 2016).

The Mind Nutrition randomized controlled trial is the first study investigating the effectiveness of nutrition counselling on depressive symptoms, depressive symptoms–related sick leave days, work ability and the quality of life among mental health professionals. The strengths of this intervention study include the randomized, controlled study design and the combination of individual and group-based nutrition counselling grounded on theoretical frameworks that support long-term lifestyle changes (Miller, 1996; Nelson, 2017; Teixeira et al., 2015). Both individual and group counselling have been effective in dietary interventions for reducing depression (Bayes et al., 2022; Jacka et al., 2017; Parletta et al., 2019).

One limitation is the lack of blinding of the participants and study staff in this type of intervention. However, this is unlikely to cause bias for the investigators, as the data come from questionnaires completed by the participants and because the intervention provider will not analyse the data. The second limitation concerns the issues related to the questionnaires, such as temporal variability and treatment expectations that can influence the responses. However, we will use validated questionnaires to minimize these issues. The third potential limitation is that subjects randomized to the control group might withdraw from the study. We try to reduce the risk by offering them one nutrition counselling session, feedback on their food records and a personal nutrition plan after the intervention. The fourth possible limitation is that the participants in both groups may modify their eating habits because they are aware of being observed, the phenomenon known as the Hawthorne effect (Sedgwick and Greenwood, 2015). Moreover, participants in the intervention and control groups may have different treatment expectancies that can influence their behavioural and self-reported outcomes.

This study will provide novel evidence of the effectiveness of nutrition counselling on depressive symptoms among mental health and substance abuse professionals. Based on the acquired knowledge, prevention programmes can be implemented for the targeted population to sustain their mental health, quality of life and work ability.

## Supplemental Material

sj-docx-1-nah-10.1177_02601060251332358 - Supplemental material for Effectiveness of improving nutrition on depressive symptoms and work ability: Study protocol for the mind nutrition randomized controlled trial Supplemental material, sj-docx-1-nah-10.1177_02601060251332358 for Effectiveness of improving nutrition on depressive symptoms and work ability: Study protocol for the mind nutrition randomized controlled trial by Aino Kipfer, Minna Kahala, Henna Kyhä, Cimmo Nurmi, Juha Puustinen, Jyrki Korkeila, Anu Ruusunen and Susanna Kunvik in Nutrition and Health

sj-docx-2-nah-10.1177_02601060251332358 - Supplemental material for Effectiveness of improving nutrition on depressive symptoms and work ability: Study protocol for the mind nutrition randomized controlled trial Supplemental material, sj-docx-2-nah-10.1177_02601060251332358 for Effectiveness of improving nutrition on depressive symptoms and work ability: Study protocol for the mind nutrition randomized controlled trial by Aino Kipfer, Minna Kahala, Henna Kyhä, Cimmo Nurmi, Juha Puustinen, Jyrki Korkeila, Anu Ruusunen and Susanna Kunvik in Nutrition and Health

sj-docx-3-nah-10.1177_02601060251332358 - Supplemental material for Effectiveness of improving nutrition on depressive symptoms and work ability: Study protocol for the mind nutrition randomized controlled trial Supplemental material, sj-docx-3-nah-10.1177_02601060251332358 for Effectiveness of improving nutrition on depressive symptoms and work ability: Study protocol for the mind nutrition randomized controlled trial by Aino Kipfer, Minna Kahala, Henna Kyhä, Cimmo Nurmi, Juha Puustinen, Jyrki Korkeila, Anu Ruusunen and Susanna Kunvik in Nutrition and Health
