# Supplementary material for: Effectiveness of improving nutrition on depressive symptoms and work ability: Study protocol for the mind nutrition randomized controlled trial
Source: Nutr Health. 2025 Apr 21;31(3):817–26. doi: 10.1177/02601060251332358 (PMC12423455; doi:10.1177/02601060251332358)
Supplement: sj-docx-1-nah-10.1177_02601060251332358 - Supplemental material for Effectiveness of improving nutrition on depressive symptoms and work ability: Study protocol for the mind nutrition randomized controlled trial [file sj-docx-1-nah-10.1177_02601060251332358.docx]

**Mind Nutrition 6-Month Survey**

The purpose of this survey is to gather information related to your mental well-being, work ability, life situation, health, and lifestyle. The survey has been developed as part of the Mind Nutrition study.

We kindly ask you to follow the instructions and answer each question accordingly.
Answering the survey will take approximately 15–30 minutes.

**Privacy policy**

By answering the survey, you give your consent for the information to be used in the Mind Nutrition project. Participation in the survey is voluntary. The data collected is confidential and will be analyzed and reported in such a way that individuals cannot be identified. Your personal information will be processed in accordance with data protection regulations, and you have the opportunity to review the project's privacy policy. More information about data security can also be found in the project’s information sheet that has been previously provided to you.

I accept the privacy policy.

- Yes
- No

**Personal Information**

In this section of the survey, we will ask for your personal information. Personal data will not be used in an identifiable form when reporting the project’s results.

1. **Identification code** (You can find this in the email sent to you. Please enter the code exactly as it appears)

**__________________________**

1. **Weight** (enter the number in kilograms, e.g. 70)
   Please provide your self-measured weight or the weight measured by a healthcare professional within the last two weeks. If you do not wish to disclose your weight, write "I do not want to disclose".

**__________________________**

**Mental Well-being**This section aims to assess your mental well-being.

Do you have a valid depression diagnosis?

- No
- Yes, I have been diagnosed during the past one year
- Yes, I have been diagnosed more than one year ago

Center for Epidemiological Studies – Depression Scale (CES-D) (Radloff, 1977)

Instructions: Please read each question carefully, then choose the option that best describes how you felt or behaved during the past week, including today.

| During the past week: | Rarely or none of the time  (less than 1 day) | Some or a little of the time (1–2 days) | Occasionally or a moderate amount of the time (3–4 days) | Most or all of the time (5–7 days) |
| --- | --- | --- | --- | --- |
| 1. I was bothered by things that usually don’t bother me |  |  |  |  |
| 1. I did not feel like eating; my appetite was poor |  |  |  |  |
| 1. I felt that I could not shake off the blues even with help from my family and friends |  |  |  |  |
| 1. I felt that I was just as good as other people |  |  |  |  |
| 1. I had trouble keeping my mind on what I was doing |  |  |  |  |
| 1. I felt depressed |  |  |  |  |
| 1. I felt that everything I did was an effort |  |  |  |  |
| 1. I felt hopeful about the future |  |  |  |  |
| 1. I thought my life had been a failure |  |  |  |  |
| 1. I felt fearful |  |  |  |  |
| 1. My sleep was restless |  |  |  |  |
| 1. I was happy |  |  |  |  |
| 1. I talked less than usual |  |  |  |  |
| 1. I felt lonely |  |  |  |  |
| 1. People were unfriendly |  |  |  |  |
| 1. I enjoyed life |  |  |  |  |
| 1. I had crying spells |  |  |  |  |
| 1. I felt sad |  |  |  |  |
| 1. I felt that people disliked me |  |  |  |  |
| 1. I could not get “going” |  |  |  |  |

**Quality of Life**WHO8-EUROHIS* (Power, 2003)

On this form, we ask you to evaluate your quality of life, health, and other aspects of your daily life. Please answer all the questions. If you are unsure about which option to choose, select the one that seems most appropriate. Often, the most suitable answer is the one that comes to mind first. When answering, think about the things you consider important, your wishes, as well as your sources of pleasure and concern. Reflect on your life over the past two weeks.

Read each question and evaluate how you feel. For each question, choose the number that you believe best corresponds to the most appropriate answer.

|  | Very bad | Bad | Neither bad nor good | Good | Very good |
| --- | --- | --- | --- | --- | --- |
| 1. How would you rate your quality of life? |  |  |  |  |  |

|  | Very dissatisfied | dissatisfied | Neither dissatisfied nor satisfied | Satisfied | Very satisfied |
| --- | --- | --- | --- | --- | --- |
| 1. How satisfied are you with your health? |  |  |  |  |  |

The following questions pertain to the extent to which you have experienced the following things during the past two weeks.

|  | Not at all | A little | Moderately | Nearly sufficiently | Fully sufficiently |
| --- | --- | --- | --- | --- | --- |
| 1. Do you have enough energy for everyday life? |  |  |  |  |  |
| 1. Have you enough money to meet your needs? |  |  |  |  |  |

In the following questions, you are asked to indicate how satisfied you have been with various aspects of your life over the past two weeks.

|  | Very dissatisfied | Dissatisfied | Neither dissatisfied nor satisfied | Satisfied | Very satisfied |
| --- | --- | --- | --- | --- | --- |
| 1. How satisfied are you with your ability to perform your daily activities? |  |  |  |  |  |
| 1. How satisfied are you with yourself? |  |  |  |  |  |
| 1. How satisfied are you with your personal relationships? |  |  |  |  |  |
| 1. How satisfied are you with the conditions of your living place? |  |  |  |  |  |

*Part of this questionnaire is translated by the authors of the Mind Nutrition study as the original, complete English questionnaire was unavailable.

**Work Ability and Well-being at Work**(Gould et al., 2015; Tuomi et al., 2007)

1. Assuming that the best working capacity you have ever had would score 10 on a scale of 0 to 10, how would you score your working capacity at present? A score of 0 would mean that you are completely unable to work at present.

________________________________________________________________________
0 = Completely unable to work Work ability at its best = 10

2. Regardless of whether you are employed or not, please estimate your current work ability.

1. Completely fit for work
2. Partially unable to work
3. Completely unable to work

3. How many sick leave days related to depressive symptoms have you had in the past six months?
*(Your response only needs to reflect your own assessment of whether your sick leave was related to depressive symptoms).*

- Not at all (0)
- 1–6 days
- 7–14 days
- 15–29 days
- 30–49 days
- 50 days or more

**Lifestyle**
In this section, we will ask you about your lifestyle and health.

1. How would you assess your current health status?

- Very poor
- Fairly poor
- Moderate
- Fairly good
- Very good

1. How many hours do you sleep on average per day? Give your answer in hours: minutes, e.g., 8:30.

__________________

1. Do you think you get enough sleep?

| 1 | 2 | 3 | 4 | 5 | 6 | 7 | 8 | 9 | 10 |
| --- | --- | --- | --- | --- | --- | --- | --- | --- | --- |

Not at all Completely sufficiently

1. How satisfied are you with your diet?

| 1 | 2 | 3 | 4 | 5 | 6 | 7 | 8 | 9 | 10 |
| --- | --- | --- | --- | --- | --- | --- | --- | --- | --- |

Very dissatisfied Very satisfied

1. How satisfied are you with your meal rhythm? *

| 1 | 2 | 3 | 4 | 5 | 6 | 7 | 8 | 9 | 10 |
| --- | --- | --- | --- | --- | --- | --- | --- | --- | --- |

Very dissatisfied Very satisfied

**Eating behavior**Three-Factor Eating Questionnaire – 18 (TFEQ-18) (Karlsson et al., 2000)

Please read each statement and select from the multiple choice options the answer that indicates the frequency with which you find yourself feeling or experiencing what is being described in the statements below.

|  | Definitely true | Mostly true | Mostly false | Definitely false |
| --- | --- | --- | --- | --- |
| 1. When I smell a delicious food, I find it very difficult to keep from eating, even if I have just finished a meal. |  |  |  |  |
| 1. I deliberately take small helpings as a means of controlling my weight. |  |  |  |  |
| 1. When I feel anxious, I find myself eating. |  |  |  |  |
| 1. Sometimes when I start eating, I just can’t seem to stop. |  |  |  |  |
| 1. Being with someone who is eating often makes me hungry enough to eat also. |  |  |  |  |
| 1. When I feel blue, I often overeat. |  |  |  |  |
| 1. When I see a real delicacy, I often get so hungry that I have to eat right away. |  |  |  |  |
| 1. I get so hungry that my stomach often seems like a bottomless pit. |  |  |  |  |
| 1. I am always hungry so it is hard for me to stop eating before I finish the food on my plate. |  |  |  |  |
| 1. When I feel lonely, I console myself by eating. |  |  |  |  |
| 1. I consciously hold back at meals in order not to weight gain |  |  |  |  |
| 1. I do not eat some foods because they make me fat. |  |  |  |  |
| 1. I am always hungry enough to eat at any time. |  |  |  |  |

|  | Only at mealtimes | Sometimes between meals | Often between meals | Almost always |
| --- | --- | --- | --- | --- |
| 1. How often do you feel hungry? |  |  |  |  |

|  | Almost never | Seldom | Moderately likely | Almost always |
| --- | --- | --- | --- | --- |
| 1. How frequently do you avoid “stocking up” on tempting foods? |  |  |  |  |

|  | Unlikely | Slightly likely | Moderately likely | Very likely |
| --- | --- | --- | --- | --- |
| 1. How likely are you to consciously eat less than you want? |  |  |  |  |

|  | Never | Rarely | Sometimes | At least once a week |
| --- | --- | --- | --- | --- |
| 1. Do you go on eating binges though you are not hungry? |  |  |  |  |

1. On a scale of 1 to 8, where 1 means no restraint in eating (eating whatever you want, whenever you want it ) and 8 means total restraint (constantly limiting food intake and never “giving in”), what number would you give yourself?

| 1 | 2 | 3 | 4 | 5 | 6 | 7 | 8 |
| --- | --- | --- | --- | --- | --- | --- | --- |

**Acceptability and Feedback**Theoretical Framework for Acceptability (TFA) (Sekhon et al., 2022)

On this form, we are collecting information about participants' experiences with the acceptability of the intervention, as well as open-ended feedback. Please read the questions and statements carefully and respond to the best of your ability.

1. How comfortable did you feel about engaging with the intervention?
   1. Very uncomfortable
   2. Uncomfortable
   3. No opinion
   4. Comfortable
   5. Very comfortable
2. How much effort did it take to participate in the intervention and complete the exercises?
   1. No effort at all
   2. A little effort
   3. No opinion
   4. A lot of effort
   5. Huge effort
3. The topics discussed in the meetings were contrary to my own values.
   1. Strongly disagree
   2. Disagree
   3. No opinion
   4. Agree
   5. Strongly agree
4. The intervention has improved my wellbeing.
   1. Strongly disagree
   2. Disagree
   3. No opinion
   4. Agree
   5. Strongly agree
5. The purpose of the intervention was clear to me.
   1. Strongly disagree
   2. Disagree
   3. No opinion
   4. Agree
   5. Strongly agree
6. How confident did you feel about applying the guidance and exercises in your daily life?
   1. Very unconfident
   2. Unconfident
   3. No opinion
   4. Confident
   5. Very confident
7. Engaging in the intervention interfered with my other priorities.
   1. Strongly disagree
   2. Disagree
   3. No opinion
   4. Agree
   5. Strongly agree
8. How acceptable was the intervention to you?
   1. Completely unacceptable
   2. Unacceptable
   3. No opinion
   4. Acceptable
   5. Completely acceptable

**Feedback**

1. On a scale of 1-10, what number would you give to individual nutrition counseling?

| 1 | 2 | 3 | 4 | 5 | 6 | 7 | 8 | 9 | 10 |
| --- | --- | --- | --- | --- | --- | --- | --- | --- | --- |

Very bad Very good

1. On a scale of 1-10, what number would you give to group nutrition counseling?

| 1 | 2 | 3 | 4 | 5 | 6 | 7 | 8 | 9 | 10 |
| --- | --- | --- | --- | --- | --- | --- | --- | --- | --- |

Very bad Very good

1. What aspects did you find beneficial in nutrition counseling?

_____________________________________________________________

_____________________________________________________________

1. What improvements do you think could be made in the overall nutrition counseling?
   **_____________________________________________________________**

**_____________________________________________________________**

**Thank you very much for your responses! If you wish, you can write feedback about the survey below.**

**______________________________________________________________**

______________________________________________________________

______________________________________________________________

______________________________________________________________

______________________________________________________________

**References**

Gould R, Härkäpää K and Koskinen S (2015) Työkyvyn arviointi väestötutkimuksessa. Finnish Institute for Health and Welfare. TOIMIA Functioning Measures Database.

Karlsson J, Persson LO, Sjöström L, et al. (2000) Psychometric properties and factor structure of the Three-Factor Eating Questionnaire (TFEQ) in obese men and women. Results from the Swedish Obese Subjects (SOS) study. International Journal of Obesity and Related Metabolic Disorders: Journal of the International Association for the Study of Obesity 24(12): 1715–1725.

Power M (2003) Development of a common instrument for quality of life. In: Nosikov A, Gudex C. EUROHIS: Developing Common Instruments for Health Surveys. Amsterdam: IOS Press, pp. 145–159.

Radloff LS (1977) The CES-D Scale: A Self-Report Depression Scale for Research in the General Population. Applied Psychological Measurement 1(3): 385–401.

Sekhon M, Cartwright M and Francis JJ (2022) Development of a theory-informed questionnaire to assess the acceptability of healthcare interventions. BMC health services research 22(1): 279.

Tuomi, K., Ilmarinen, J., Jahkola, A., Katajarinne, L., Tulkki, A. 2007. The Work Ability Index (WAI). Occup. Med. 57, 160–160. https://doi.org/10.1093/occmed/kqm008
