# Supplementary material for: Effectiveness of improving nutrition on depressive symptoms and work ability: Study protocol for the mind nutrition randomized controlled trial
Source: Nutr Health. 2025 Apr 21;31(3):817–26. doi: 10.1177/02601060251332358 (PMC12423455; doi:10.1177/02601060251332358)
Supplement: sj-docx-3-nah-10.1177_02601060251332358 - Supplemental material for Effectiveness of improving nutrition on depressive symptoms and work ability: Study protocol for the mind nutrition randomized controlled trial [file sj-docx-3-nah-10.1177_02601060251332358.docx]

**Food Frequency Questionnaire**

**The purpose of this survey is to gather information about your eating habits. When filling out this form, please think about the past 12 months. If you do not prepare or purchase food, it is a good idea to complete the survey together with the person who does the cooking in your household. Before you start filling out the form, take a moment to review it by looking through the questions.**

**In the following questions that describe your eating habits, circle the one option that best fits, except for question 24, where you may select as many options as necessary.**

**24. Which of the following meals do you eat on a typical day (circle):**
Breakfast Yes No
Morning snack Yes No
Lunch Yes No
Afternoon snack/coffee Yes No
Dinner Yes No
Snack Yes No
Evening snack Yes No
Late-night snack Yes No

**25. Where do you eat your warm meals on a regular weekday?**

1. At home or homemade food elsewhere (e.g., packed lunch)
2. Outside the home (e.g., workplace cafeteria or restaurant)
3. Both homemade and outside-prepared food

**26. What kind of fat do you usually use in cooking at home?**

1. No cooking at home
2. Butter, Oivariini, or a similar product containing dairy fat
3. Spreadable margarine (e.g., Becel, Flora)
4. Liquid vegetable fat (e.g., Keiju, Flora liquid, Flora Culinesse)
5. Baking margarine (e.g., Milda, Sunnuntai)
6. Cooking oil
7. No fat is usually used in cooking

**27. What kind of salad dressing do you usually choose?**

1. I don’t use salad dressings
2. Oil-based salad dressing or oil
3. Sour cream-based dressing
4. Mayonnaise-based dressing
5. Juice
6. Fat-free dressings

**28. What kind of fat do you usually use in baking?**

1. No baking at home
2. Butter, Oivariini, or similar (mix of butter and vegetable oil)
3. Spreadable margarine
4. Liquid vegetable fat (bottle margarine)
5. Baking margarine (block margarine, e.g., Milda, Sunnuntai)
6. Cooking oil

**29. How often do you eat fried foods in fat?**

1. Daily
2. 4–6 times a week
3. 1–3 times a week
4. Less than once a week

**30. How often do you eat breakfast within one hour of waking up?**

1. Daily
2. 5–6 times a week
3. 3–4 times a week
4. 1–2 times a week
5. Less often or never

**How often do you usually eat the following foods? Circle the most appropriate option for frequency of consumption. If your typical portion size is smaller than the average portion listed on the form, you can adjust your frequency estimate to slightly lower. Similarly, if your portion size is larger than the mentioned portion size, consider that by estimating your frequency higher.**

**IF YOU DO NOT CONSUME THE PRODUCT, PLEASE CIRCLE "NOT AT ALL, RARELY" THROUGHOUT THE FORM.**

**DAIRY PRODUCTS**

**Average consumption over the past 12 months (circle 1 option)**
Note: Coffee milk is asked later

|  | Not at all, rarely | 1–3 times /month | Once /week | 5–6 times /week | Once /day | 2–3 times /day | 4–5 times /day | ≥ 6 times /day |
| --- | --- | --- | --- | --- | --- | --- | --- | --- |
| 31. Whole milk (glass, 1.7 dl) | 0 | 1 | 2 | 3 | 4 | 5 | 6 | 7 |
| 32. Low fat (1.5%) milk (glass) | 0 | 1 | 2 | 3 | 4 | 5 | 6 | 7 |
| 33. 1% milk (glass) | 0 | 1 | 2 | 3 | 4 | 5 | 6 | 7 |
| 34. Fat-free milk (glass | 0 | 1 | 2 | 3 | 4 | 5 | 6 | 7 |
| 35. Organic fat free milk (glass) | 0 | 1 | 2 | 3 | 4 | 5 | 6 | 7 |
| 36. Organic low fat (1.5%) milk (glass) | 0 | 1 | 2 | 3 | 4 | 5 | 6 | 7 |
| 37. Soy, oat, or rice milk (glass) | 0 | 1 | 2 | 3 | 4 | 5 | 6 | 7 |
| 38. Buttermilk, fat free (glass, 1.7 dl) | 0 | 1 | 2 | 3 | 4 | 5 | 6 | 7 |
| 39. Organic butter milk (glass) | 0 | 1 | 2 | 3 | 4 | 5 | 6 | 7 |
| 40. Buttermilk, other (glass) | 0 | 1 | 2 | 3 | 4 | 5 | 6 | 7 |
| 41. Yogurt or nordic kind of sour milk, fat free (2 dl) | 0 | 1 | 2 | 3 | 4 | 5 | 6 | 7 |
| 42. Yogurt or sour milk, other (2 dl) | 0 | 1 | 2 | 3 | 4 | 5 | 6 | 7 |
| 43. Cheese, high fat (> 26 %), e.g. Emmental, Turunmaa (2 slices=20 g) | 0 | 1 | 2 | 3 | 4 | 5 | 6 | 7 |
| 44. Cheese, middle fat (18–26%), e.g. Edam (2 slices=20 g) | 0 | 1 | 2 | 3 | 4 | 5 | 6 | 7 |
| 45. Cheese, low fat (10–17%), e.g. Oltermanni 17% (20 g) | 0 | 1 | 2 | 3 | 4 | 5 | 6 | 7 |
| 46. Cheese, fat content less than 10%, e.g. Polar 5% (20g) | 0 | 1 | 2 | 3 | 4 | 5 | 6 | 7 |
| 47. Quark (1 dl) | 0 | 1 | 2 | 3 | 4 | 5 | 6 | 7 |
| 48. Cottage cheese (1 dl) | 0 | 1 | 2 | 3 | 4 | 5 | 6 | 7 |
| 49. Cooking cream (1 dl) | 0 | 1 | 2 | 3 | 4 | 5 | 6 | 7 |
| 50. Cream-like vegetable product used in cooking (1 dl) | 0 | 1 | 2 | 3 | 4 | 5 | 6 | 7 |
| **GRAIN PRODUCTS** | | | | | | | | |
| 51. Rye bread (1 slice, 30 g) | 0 | 1 | 2 | 3 | 4 | 5 | 6 | 7 |
| 52. Mixed bread (1 slice, 30 g) | 0 | 1 | 2 | 3 | 4 | 5 | 6 | 7 |
| 53. French bread, baguette, or other completely white bread (1 slice or piece, 30 g) | 0 | 1 | 2 | 3 | 4 | 5 | 6 | 7 |
| 54. Crispbread, sourdough crispbread (1 slice) | 0 | 1 | 2 | 3 | 4 | 5 | 6 | 7 |
| 55. Porridge or thin gruel (2 dl) | 0 | 1 | 2 | 3 | 4 | 5 | 6 | 7 |
| 56. Breakfast cereals (2 dl) | 0 | 1 | 2 | 3 | 4 | 5 | 6 | 7 |
| 57. Muesli (1 dl) | 0 | 1 | 2 | 3 | 4 | 5 | 6 | 7 |
| 58. Bun or bun-based pastry, e.g. rusk or blueberry pie (1 piece) | 0 | 1 | 2 | 3 | 4 | 5 | 6 | 7 |
| 59. Danish pastry or donut (1 piece) | 0 | 1 | 2 | 3 | 4 | 5 | 6 | 7 |
| 60. Cake, Swiss roll, or pastry (portion or 1 piece) | 0 | 1 | 2 | 3 | 4 | 5 | 6 | 7 |
| 61. Savory biscuit, e.g. Cream cracker (2 pieces) | 0 | 1 | 2 | 3 | 4 | 5 | 6 | 7 |
| 62. Sweet cookie or biscuit (1 piece) | 0 | 1 | 2 | 3 | 4 | 5 | 6 | 7 |
| 63. Karelian pie (1 piece, 65 g) | 0 | 1 | 2 | 3 | 4 | 5 | 6 | 7 |
| 64. Savory pastry or pie (1 piece, 65 g) | 0 | 1 | 2 | 3 | 4 | 5 | 6 | 7 |
| **BREAD SPREADS** | | | | | | | | |
| 65. Butter, Oivariini, or other butter-oil mixture (5 g) | 0 | 1 | 2 | 3 | 4 | 5 | 6 | 7 |
| 66. Packet margarine, 60–70 % fat (5 g) | 0 | 1 | 2 | 3 | 4 | 5 | 6 | 7 |
| 67. Packet margarine, 20–40 % fat (5 g) | 0 | 1 | 2 | 3 | 4 | 5 | 6 | 7 |
| 68. Spread with plant stanol esters (Benecol / Becel Pro activ / Keiju alentaja, 5 g) | 0 | 1 | 2 | 3 | 4 | 5 | 6 | 7 |
| 69. Processed or cream cheese, fat > 20% (e.g. Olympia, Koskenlaskija, 5 g) | 0 | 1 | 2 | 3 | 4 | 5 | 6 | 7 |
| 70. Processed or cream cheese, fat less than 20% (e.g. Polar spreadable or Pirkka light processed cheese, 5 g) | 0 | 1 | 2 | 3 | 4 | 5 | 6 | 7 |
| **MEAT, CHICKEN, EGGS, TOFU** | | | | | | | | |
| 71. Cured sausage, salami (2 slices) | 0 | 1 | 2 | 3 | 4 | 5 | 6 | 7 |
| 72. Cold cuts sausage (2 slices) | 0 | 1 | 2 | 3 | 4 | 5 | 6 | 7 |
| 73. Liver sausage (1 cm slice | 0 | 1 | 2 | 3 | 4 | 5 | 6 | 7 |
| 74. Whole meat cold cut, e.g., salted beef or boiled ham (2 slices) | 0 | 1 | 2 | 3 | 4 | 5 | 6 | 7 |
| 75. Ground meat dish, e.g., meatballs or meat sauce (1 portion) | 0 | 1 | 2 | 3 | 4 | 5 | 6 | 7 |
| 76. Whole meat dish, e.g., steak, roast, or meat sauce (1 portion) | 0 | 1 | 2 | 3 | 4 | 5 | 6 | 7 |
| 77. Chicken, turkey, or other poultry dish (1 portion) | 0 | 1 | 2 | 3 | 4 | 5 | 6 | 7 |
| 78. Sausage dish, such as hot dogs or oven sausage (1 portion) | 0 | 1 | 2 | 3 | 4 | 5 | 6 | 7 |
| 79. Soup, e.g., meat or pea soup (3 dl) | 0 | 1 | 2 | 3 | 4 | 5 | 6 | 7 |
| 80. Liver dish (1 portion) | 0 | 1 | 2 | 3 | 4 | 5 | 6 | 7 |
| 81. Blood dish (1 portion) | 0 | 1 | 2 | 3 | 4 | 5 | 6 | 7 |
| 82. Bacon (5 slices, approximately 100 g) | 0 | 1 | 2 | 3 | 4 | 5 | 6 | 7 |
| 83. Pizza (300 g portion) | 0 | 1 | 2 | 3 | 4 | 5 | 6 | 7 |
| 84. Hamburger (regular size, about 150 g) | 0 | 1 | 2 | 3 | 4 | 5 | 6 | 7 |
| 85. Egg (1 piece) | 0 | 1 | 2 | 3 | 4 | 5 | 6 | 7 |
| 86. Tofu (100 g) | 0 | 1 | 2 | 3 | 4 | 5 | 6 | 7 |
| **FISH** | | | | | | | | |
| Fish dish from the following fish: | | | | | | | | |
| 87. Salmon, rainbow trout, trout, char, sardine, or mackerel, baked, fried, etc. (portion=3 dl) | 0 | 1 | 2 | 3 | 4 | 5 | 6 | 7 |
| 1. Vendace, bream, whitefish, roach, or Baltic herring, baked, fried, etc. (1 portion or 1 can, approx. 150 g) | 0 | 1 | 2 | 3 | 4 | 5 | 6 | 7 |
| 1. Pollock, perch, pikeperch, burbot, or pike, baked, fried, etc. (1 portion or 1 can, approx. 150 g) | 0 | 1 | 2 | 3 | 4 | 5 | 6 | 7 |
| 1. Fish soup from salmon or rainbow trout (portion = 3 dl) | 0 | 1 | 2 | 3 | 4 | 5 | 6 | 7 |
| 1. Fish soup from other fish (portion = 3 dl) | 0 | 1 | 2 | 3 | 4 | 5 | 6 | 7 |
| 1. Sardine or mackerel can (1 can = approx. 100 g) | 0 | 1 | 2 | 3 | 4 | 5 | 6 | 7 |
| 1. Tuna in oil (½ can = 70 g) | 0 | 1 | 2 | 3 | 4 | 5 | 6 | 7 |
| 1. Tuna in water (½ can = 70 g) | 0 | 1 | 2 | 3 | 4 | 5 | 6 | 7 |
| 1. Herring (2 slices) | 0 | 1 | 2 | 3 | 4 | 5 | 6 | 7 |
| 1. Other seasoned or salted fish, such as gravlax or anchovies (2 slices) | 0 | 1 | 2 | 3 | 4 | 5 | 6 | 7 |
| 1. Kalakukko (Finnish fish pastry, 200 g) | 0 | 1 | 2 | 3 | 4 | 5 | 6 | 7 |
| 1. Shrimp, mussels, shellfish (1 dl) | 0 | 1 | 2 | 3 | 4 | 5 | 6 | 7 |
| **VEGETABLES** | | | | | | | | |
| 1. Fresh salads (1.5 dl) | 0 | 1 | 2 | 3 | 4 | 5 | 6 | 7 |
| 1. Grated vegetables (1.5 dl) | 0 | 1 | 2 | 3 | 4 | 5 | 6 | 7 |
| 1. Mayonnaise-based salad, such as potato or beetroot salad (1 dl) | 0 | 1 | 2 | 3 | 4 | 5 | 6 | 7 |
| 1. Raw root vegetables (1 carrot or equivalent) | 0 | 1 | 2 | 3 | 4 | 5 | 6 | 7 |
| 1. Tomato (1 piece) | 0 | 1 | 2 | 3 | 4 | 5 | 6 | 7 |
| 1. Cucumber (2 cm slice) | 0 | 1 | 2 | 3 | 4 | 5 | 6 | 7 |
| 1. Cauliflower, fresh (1 dl) | 0 | 1 | 2 | 3 | 4 | 5 | 6 | 7 |
| 1. Bell pepper (2 slices) | 0 | 1 | 2 | 3 | 4 | 5 | 6 | 7 |
| 1. Beetroot, boiled (1 dl) | 0 | 1 | 2 | 3 | 4 | 5 | 6 | 7 |
| **COOKED VEGETABLES** | | | | | | | | |
| 1. Mixed vegetables, soup vegetables, stir-fry vegetables (1 dl) | 0 | 1 | 2 | 3 | 4 | 5 | 6 | 7 |
| 1. Peas and beans (1 dl) | 0 | 1 | 2 | 3 | 4 | 5 | 6 | 7 |
| 1. Cauliflower (1 dl) | 0 | 1 | 2 | 3 | 4 | 5 | 6 | 7 |
| 1. Asparagus or Brussels sprouts (1 dl) | 0 | 1 | 2 | 3 | 4 | 5 | 6 | 7 |
| 1. Carrot (1 dl) | 0 | 1 | 2 | 3 | 4 | 5 | 6 | 7 |
| 1. Cabbage casserole, soup, or rolls (1 portion) | 0 | 1 | 2 | 3 | 4 | 5 | 6 | 7 |
| 1. Other vegetable dishes, such as puree soup (1 portion) | 0 | 1 | 2 | 3 | 4 | 5 | 6 | 7 |
| 1. Mushrooms (1 dl) | 0 | 1 | 2 | 3 | 4 | 5 | 6 | 7 |
| **POTATO, RICE, PASTA** | | | | | | | | |
| 1. Boiled or baked potato or mashed potatoes (2 pieces or 2 dl) | 0 | 1 | 2 | 3 | 4 | 5 | 6 | 7 |
| 1. Fried or French fries (2 dl) | 0 | 1 | 2 | 3 | 4 | 5 | 6 | 7 |
| 1. Potato-based casseroles or gratins (1 portion) | 0 | 1 | 2 | 3 | 4 | 5 | 6 | 7 |
| 1. Macaroni-based casseroles or lasagna (1 portion) | 0 | 1 | 2 | 3 | 4 | 5 | 6 | 7 |
| 1. Boiled rice or risotto, rice noodles (1 portion) | 0 | 1 | 2 | 3 | 4 | 5 | 6 | 7 |
| 1. Spaghetti or other pasta products such as wheat noodles (1 portion) | 0 | 1 | 2 | 3 | 4 | 5 | 6 | 7 |
| **FRUITS AND BERRIES** | | | | | | | | |
| 1. Apple (1 piece) | 0 | 1 | 2 | 3 | 4 | 5 | 6 | 7 |
| 1. Orange, grapefruit (1 piece) | 0 | 1 | 2 | 3 | 4 | 5 | 6 | 7 |
| 1. Mandarin, satsuma, etc. (2 pieces) | 0 | 1 | 2 | 3 | 4 | 5 | 6 | 7 |
| 1. Banana ( 1 piece) | 0 | 1 | 2 | 3 | 4 | 5 | 6 | 7 |
| 1. Grapes (10 pieces) | 0 | 1 | 2 | 3 | 4 | 5 | 6 | 7 |
| 1. Avocado (1 piece) | 0 | 1 | 2 | 3 | 4 | 5 | 6 | 7 |
| 1. Other such as pear, kiwi or melon (1 piece or 100 g) | 0 | 1 | 2 | 3 | 4 | 5 | 6 | 7 |
| 1. Fresh or frozen berries (1 dl) | 0 | 1 | 2 | 3 | 4 | 5 | 6 | 7 |
| 1. Berry compote (1 dl) | 0 | 1 | 2 | 3 | 4 | 5 | 6 | 7 |
| 1. Berry or fruit curd (1 dl) | 0 | 1 | 2 | 3 | 4 | 5 | 6 | 7 |
| 1. Diluted berry juice (glass, 1.7 dl) | 0 | 1 | 2 | 3 | 4 | 5 | 6 | 7 |
| 1. Orange juice (glass) | 0 | 1 | 2 | 3 | 4 | 5 | 6 | 7 |
| 1. Apple juice (glass) | 0 | 1 | 2 | 3 | 4 | 5 | 6 | 7 |
| 1. Other fruit juice (glass) | 0 | 1 | 2 | 3 | 4 | 5 | 6 | 7 |
| 1. Jam or marmalade (1 tbsp) | 0 | 1 | 2 | 3 | 4 | 5 | 6 | 7 |
| 1. Salt liquorice (1 pack or 40 g loose) | 0 | 1 | 2 | 3 | 4 | 5 | 6 | 7 |
| 1. Licorice (6 pieces or 1 bar, 20 g) | 0 | 1 | 2 | 3 | 4 | 5 | 6 | 7 |
| 1. Caramel (e.g. loose candy, 100 g) | 0 | 1 | 2 | 3 | 4 | 5 | 6 | 7 |
| 1. Ice cream or pudding (1.5 dl) | 0 | 1 | 2 | 3 | 4 | 5 | 6 | 7 |
| 1. Chocolate (1 large bar or 45 g) | 0 | 1 | 2 | 3 | 4 | 5 | 6 | 7 |
| **DRINKS, SWEETENERS, ETC.** | | | | | | | | |
| 1. Filter coffee (1 cup, 1.5 dl) | 0 | 1 | 2 | 3 | 4 | 5 | 6 | 7 |
| 1. Pot coffee or other coffee made without a filter paper (1 cup) | 0 | 1 | 2 | 3 | 4 | 5 | 6 | 7 |
| 1. Instant coffee (1 cup) | 0 | 1 | 2 | 3 | 4 | 5 | 6 | 7 |
| 1. Tea (1 cup, 1.5 dl) | 0 | 1 | 2 | 3 | 4 | 5 | 6 | 7 |
| 1. Herbal tea, such as chamomile tea (1 cup) | 0 | 1 | 2 | 3 | 4 | 5 | 6 | 7 |
| 1. Cocoa (1 cup) | 0 | 1 | 2 | 3 | 4 | 5 | 6 | 7 |
| 1. Milk in coffee or tea (1 tbsp) | 0 | 1 | 2 | 3 | 4 | 5 | 6 | 7 |
| 1. Sugar in coffee or tea (2 cubes) | 0 | 1 | 2 | 3 | 4 | 5 | 6 | 7 |
| 1. Cream in coffee or tea (1 tbsp) | 0 | 1 | 2 | 3 | 4 | 5 | 6 | 7 |
| 1. Sweetener in drinks or otherwise, such as saccharin | 0 | 1 | 2 | 3 | 4 | 5 | 6 | 7 |
| 1. Honey (1 tbsp = 20 g) | 0 | 1 | 2 | 3 | 4 | 5 | 6 | 7 |
| 1. Sugared soft drinks or energy drinks (0.5 l) | 0 | 1 | 2 | 3 | 4 | 5 | 6 | 7 |
| 1. Unsweetened soft drinks (0.5 l) | 0 | 1 | 2 | 3 | 4 | 5 | 6 | 7 |
| 1. Non-alcoholic beer, home-brewed beer or pilsner (0.33 l) | 0 | 1 | 2 | 3 | 4 | 5 | 6 | 7 |
| 1. Low-alcohol beer (≤ 3.5% alcohol, portion 0.5 l) | 0 | 1 | 2 | 3 | 4 | 5 | 6 | 7 |
| 1. Medium-alcohol beer (≤ 4.7% alcohol, 0.5 l) | 0 | 1 | 2 | 3 | 4 | 5 | 6 | 7 |
| 1. Strong beer (5.5 % alcohol, 0.5 l) | 0 | 1 | 2 | 3 | 4 | 5 | 6 | 7 |
| 1. Long drink (0.5 l) | 0 | 1 | 2 | 3 | 4 | 5 | 6 | 7 |
| 1. Long drink, artificially sweetened, light (0.5 l) | 0 | 1 | 2 | 3 | 4 | 5 | 6 | 7 |
| 1. Cider (0.5 l) | 0 | 1 | 2 | 3 | 4 | 5 | 6 | 7 |
| 1. Cider, artificially sweetened, light (0.5 l) | 0 | 1 | 2 | 3 | 4 | 5 | 6 | 7 |
| 1. Red wine (restaurant portion, 12 cl = 1.2 dl) | 0 | 1 | 2 | 3 | 4 | 5 | 6 | 7 |
| 1. White wine (restaurant portion, 12 cl = 1.2 dl) | 0 | 1 | 2 | 3 | 4 | 5 | 6 | 7 |
| 1. Other wine (sparkling wine, rosé wine, restaurant portion, 1.2 dl) | 0 | 1 | 2 | 3 | 4 | 5 | 6 | 7 |
| 1. Fortified wine (liqueur, sherry, port, etc., restaurant portion 8 cl) | 0 | 1 | 2 | 3 | 4 | 5 | 6 | 7 |
| 1. Spirits or other strong alcohol (restaurant portion 4 cl) | 0 | 1 | 2 | 3 | 4 | 5 | 6 | 7 |
| **OTHER FOODS** | | | | | | | | |
| 1. Pickled cucumber (5 slices) | 0 | 1 | 2 | 3 | 4 | 5 | 6 | 7 |
| 1. Salted nuts or seeds (50 g) | 0 | 1 | 2 | 3 | 4 | 5 | 6 | 7 |
| 1. Unsalted nuts or seeds (50 g) | 0 | 1 | 2 | 3 | 4 | 5 | 6 | 7 |
| 1. Potato chips, popcorn, or similar (2 dl, about 20 g) | 0 | 1 | 2 | 3 | 4 | 5 | 6 | 7 |
| 1. Olives (5 pieces) | 0 | 1 | 2 | 3 | 4 | 5 | 6 | 7 |
| 1. Foods containing lactic acid bacteria, such as Gefilus, Rela, or Actimel yogurts or drinks* | 0 | 1 | 2 | 3 | 4 | 5 | 6 | 7 |

*The use of these products should also be marked, for example, in the yogurt sections 41-42 and in the juice sections 132-135 on the form.

**USE OF SALT (choose the option that best describes you)**

1. When eating out, does the food generally taste:
2. Less salty than your home-cooked food
3. As salty as your home-cooked food
4. More salty than your home-cooked food
5. In your opinion, are ready-made meals (store-bought ready-to-eat foods) compared to homemade food:
6. Less salty than your home-cooked food
7. As salty as your home-cooked food
8. More salty than your home-cooked food
9. I don’t use ready-made meals
10. Do you add salt to the food already on your plate while eating:
11. Never or very rarely
12. Sometimes, after tasting first
13. Often, after tasting first
14. Often, without tasting first
15. What type of salt do you typically use at home:
16. Regular iodized table salt (sodium chloride), e.g. iodized Jozo®
17. Non-iodized salt (e.g. rock salt, pink Himalayan salt)
18. Sea salt
19. Mineral salt, where some of the sodium is replaced with potassium and magnesium, such as PANSUOLA® or Seltin®
